# Supplementary material for: Emergence and intensification of dairying in the Caucasus and Eurasian steppes
Source: Nat Ecol Evol. 2022 Apr 7;6(6):813–22. doi: 10.1038/s41559-022-01701-6 (PMC9177415; doi:10.1038/s41559-022-01701-6)
Supplement: Supplementary file 1 — Supplementary Discussion. [file 41559_2022_1701_MOESM1_ESM.pdf]

nature portfolio

https://doi.org/10.1038/s41559-022-01701-6

Supplementary information

Emergence and intensification of dairying in the Caucasus and Eurasian steppes

In the format provided by the authors and unedited

Supplementary Information for

Emergence and intensification of dairying in the Caucasus and Eurasian steppes

Ashley Scott, Sabine Reinhold, Taylor Hermes, Alexey A. Kalmykov, Andrey Belinskiy, Alexandra Buzhilova, Natalia Berezina, Anatoliy R. Kantorovich, Vladimir E. Maslov, Farhad Guliyev, Bertille Lyonnet, Parviz Gasimov, Tufan Axhundov, Bakhtiyar Jalilov, Jeyhun Eminli, Emil Iskandarov, Emily Hammer, Selin Nugent, Richard Hagan, Kerttu Majander, Päivi Onkamo, Kerkko Nordqvist, Natalia Shishlina, Elena Kaverzneva, Arkadiy I. Korolev, Aleksandr A. Khokhlov, Roman V. Smolyaninov, Rüdiger Krause, Eliza Stolarczyk, Maria Karapetian, Svetlana V. Sharapova, Johannes Krause, Svend Hansen*, Wolfgang Haak*, Christina Warinner*

Correspondence to:

Christina Warinner	Wolfgang Haak	Svend Hansen
warinner@fas.harvard.edu	wolfgang_haak@eva.mpg.de	svend.hansen@dainst.de

This PDF file includes

Supplementary Discussion

1 Archaeological Site and Sample Information for North Caucasus……………………3
- 1.1 Aygursky 2 ……………………………………………………………3
- 1.2 Ilinsky 1 ……………………………………………………………4
- 1.3 Ipatovo 3 ……………………………………………………………4
- 1.4 Kabardinksky 9………………………………………………………5
- 1.5 Komsomolec 1-Marfa…………………………………………………6
- 1.6 Krasnogvardeyskoe…………………………………………………7
- 1.7 Kurganny 1……………………………………………………………7
- 1.8 Marinskaya 5…………………………………………………………8
- 1.9 Nevinnomysky 3………………………………………………………10
- 1.10 Progress 2……………………………………………………………11
- 1.11 Rasshevatsky 1………………………………………………………12
- 1.12 Rasshevatsky 4………………………………………………………13
- 1.13 Sharakhalsun 6………………………………………………………13
- 1.14 Tipki 1…………………………………………………………………15
- 1.15 Vinsadsky 2……………………………………………………………15
- 1.16 Zolotarevka 1…………………………………………………………16
- 1.17 Zolotarevka 2…………………………………………………………17

2 Archaeological Site and Sample Information for South Caucasus……………………17
- 2.1 Alkhantepe……………………………………………………………17
- 2.2 Göytepe ………………………………………………………………18
- 2.3 Qabala …………………………………………………………………19
- 2.4 Qızqala…………………………………………………………………22
- 2.5 Mentesh Tepe…………………………………………………………23
- 2.6 Uzun Rama……………………………………………………………24

3 Archaeological Site and Sample Information for Oka-Don-Volga region…………..24

3.1 Ksizovo 6……………………………………………..……..…………...24
3.2 Rakovka…………………………………………….……..…………...25
3.3 Shagara……………………………………………..……...…………...26
3.4 Vasilevsky, Kordon 27………………………………...…………..27
3.5 Rovenka…………………………………………….……..…………...28
4 Archaeological Site and Sample Information for East Urals region………………...29
4.1 Neplyuyevka ……………………………………..………...…………..29

References for SI citations ……………………………………………………...30

1. Archaeological Site and Sample Information for North Caucasus

1.1 Aygursky 2

Country: Russian Federation
Region: Apanasenkovo district, Stavropol region
Coordinates: 45.690°, 43.262°
Excavation details: Excavation 'Nasledie' 1998-2001, Stavropol, license №1998-176, №1999-804, №2000-776 & №2001-776 (V. A. Babenko)

Summary:
The Aygursky 2 burial mound cemetery is situated on a promontory overlooking the small river of Aygurky, about 22 km east of the city of Ipatovo. Excavation of the cemetery took place from 1998-2001 by the local heritage organization 'Nasledie'. In total, there were 37 burial mounds recorded at Aygursky 2. Individuals sampled for this study were from mounds 22 and 37. Mound 22 was the largest in the cemetery, with a diameter of 38-42 m and a height of 1.8 m. It included three construction phases [1]. Individual AY2002 included in this study originates from the first construction phase dated to the Maykop period. Graves 8 and 8A (AY2002) were the founding burials from the first construction phase. Other Maykop individuals from Mound 22 were part of a more extensive paleoanthropological study [2]. They were also investigated regarding their radiocarbon and stable isotope composition [3]. The stable isotope data of the fauna overlaps with that of the dry-steppe group of sites such as Sharakhalsun 6. A dietary offset has been observed when comparing radiocarbon dates of human and animal bones (see comments below) but confirmed a mid-to-late Maykop date for all graves. The presence of domesticated animals, including sheep/goats, horses, and ass, suggests a pastoralist aspect to the subsistence economy. The particularities of mortuary inventories and grave construction, together with the site's location in the grass-steppe zone, associate these complexes with a Steppe-Maykop cultural affiliation. Genome-wide genetic data have been published for two individuals from Kurgan 22 Grave 9 (AY2003) and Grave 15 (AY2001), confirming an ancestry consistent with Steppe Maykop groups [4]. Mound 37 is situated approximately 50 m northeast of Mound 22 at the slope of the watershed. The low mound was built on top of a catacomb grave inside a rectangular ditch construction, and the ditch was partly covered with stones.

- **AY2002**, AYG2/K22/G8, BZNK-0288, Kurgan 22, Grave 8A, was the burial of a child associated with the *Late Steppe Maykop culture.* Grave 8 and 8A were central to a megalithic construction combining a massive stone-box grave atop a low stone mound-shell and a surrounding stone circle. The burial of a child (Grave 8A) destroyed the previous grave (Grave 8) of an adult (age 25-25 years), and only a few bone fragments were preserved. No inventory was found in the grave beside the bone of an ovicaprid. This individual was in a crouched position on her right side with her head directed to the east-northeast. Red ochre was observed on the bones, particularly around the skull. Dating: Human Bone of the individual in Grave 8, 3769-3641

calBCE (OxA-16134, 4919±31 BP); animal bone associated with Grave 8A 3605-3369 calBCE (OxA-16135: 4688±31 BP) [3].

- **AY2005**, AYG2/K37/G1, BZNK-1013, Kurgan 37, Grave 1. This burial of an adult male is associated with the *Lola/post-Catacomb* cultural complex. The burial consisted of a well-preserved single inhumation in a catacomb with a broad entrance shaft. The individual was in a crouched position on the left side, with the head oriented towards the southeast. The burial inventory included a complete ceramic vessel, fragments of a second in the fill, a bronze dagger and an awl in front of the arms, and two possible whetstones at the feet. Dating: human bone 2196-1986 calBCE (MAMS 45944, 3702±21 BP; dating this study).

1.2 Ilinsky 1

Country: Russian Federation
Region: Izobilniy district, Stavropol region
Coordinates: 45.563379°, 41.822599°
Excavation details: Excavation 'Nasledie', Stavropol 1998, license №1998-550 (Ya. B. Berezin)

Summary:
The burial mound cemetery is situated on the terrace of the river Egorlyk, 3.9 km south-southwest of the village of Ilinskiy. Prior to excavation, burial Mounds 1, 2, and 4 were located on a meadow, while Mounds 3, 5-7 were located on arable land and affected by plowing. The cemetery consisted of southern and northern barrow clusters, approximately 0.4-0.6 km apart. Before excavation, Mound 1, the largest of the group, had a height of 2.01-1.40 m and a diameter of 50 m. At least 14 burials can be associated with two building phases, and one ritual complex was excavated. The founding grave (Grave 5) in the center dates to the beginning of the Middle Bronze Age and is one of the few complexes with a post-Maykop shaft-hole axe. The other graves in the mounds belong to various Bronze Age groups, primarily Catacomb and post-Catacomb cultures.

- **ILL001**, ILI1/K1/G6, BZNK-1026, Kurgan 1, Grave 6. This grave was associated with the *Lola/post-Catacomb culture*. The burial consisted of a single inhumation without recognizable traces of a grave-pit. A poorly preserved male skeleton was placed in a crouched position on the left side, with the head towards the southeast. There was a bronze knife in the right hand and a bone pendant at this side of the body. Dating: human bone 2276-2044 calBCE (MAMS-42350, 3751±18 BP; dating this study).

1.3 Ipatovo 3

Country: Russian Federation
Region: Ipatovo district, Stavropol region
Coordinates: 45.685°, 42.921°
Excavation details: Excavation 'Nasledie', Stavropol 1998-1999, license №1998-177, №1999-37 (A. B. Belinskiy).

Summary:
Ipatovo 3, Mound 2 was a part of a more extensive linear series of mounds that ran west-east across the Kalaus Rover, including 11 visible from aerial photos. Mound 2 was the largest and yielded 195 burials in 11 construction phases [5]. The mound construction and 34 of its burials occurred during the Bronze Age [5]. Several of the Catacomb epoch interments were accompanied by wooden wagons [6]. The first three mound-shells are associated with graves belonging to an Eneolithic or Steppe-Maykop tradition, including Grave 195 in this study. The isotopic composition of the Early Bronze Age individuals from the Ipatovo Mound 2 reflects a mixed diet of animal-derived food and terrestrial C_3 plants, with minor contributions from C_4 plants [3]. This is characteristic of a pastoralist economy, yet the mobility ranges of these individuals were limited [7]. Ovicaprid and cattle bones were placed in the graves, suggesting an important place for these animals in the local subsistence economy. The first burial in this mound, Grave 187 (IV3002), was recently identified as infected with a human-adapted form of *Salmonella enterica* at the time of death [8].

- **IV3012**, IPA3/K2/G195/I1, BZNK-1010, Kurgan 2, Grave 195, Individual 1, *Late Steppe Maykop*. The burial consisted of double inhumation. A young male (IV3012), aged 18-19 years, was buried with a teenage female sitting inside a stone-box grave with a dome-shaped cover of stone slabs. The chamber walls included one anthropomorphic slab. Burial inventory included ovicaprid bones and pottery. Dating: animal bone, 3631-3133 calBCE (KI-14218, 4650±60 BP)[9].

1.4 Kabardinsky 9
Country: Russian Federation
Region: Kislovodsk district, Stavropol region
Coordinates: 43.826°, 42.716°
Excavation details: Excavation 'Nasledie', Stavropol and Eurasia-Department DAI, Berlin 2011, license №2011-50 (A. B. Belinskiy)

Summary:
The mound cemetery of Kabardinsky 9 (also known as Kabardinka) is situated in the mountain region of the North Caucasus. The site's location is a mid-elevation mountain environment at the edge of a plateau overlooking the mineral spa of Kislovodsk. In 2011 several features in a necropolis of about a dozen small barrows and stone circles were excavated following a large-scale magnetometric survey. The excavation was part of a long-term archaeological investigation in the nearby settlement of Kabardinka 2 and related sites. The excavations targeted a typical North Caucasus Culture burial mound (Mound 1) with 13 or 14 former interments, two burials in a large and a small stone circle (Mound 2), without a mound-shell. Excavations also focused on two flat graves outside the mound group: Grave 1 and Grave 2. Central Grave 1b in Mound 2, which was sampled for this study, was surrounded by a stone circle without a mound and located 72 m west of Mound 1. The economy of the Kabardinsky 9 community was most likely based on herding [10], and ovicaprid remains were recovered from the graves. The isotopic composition of the recovered

human remains suggests an agropastoralist subsistence economy [7]. However, the relative dietary contributions of plant and animal protein cannot yet be determined.

- **KBD001**, KAB/K2/G1b, BZNK-123, Kurgan 2, Grave 1b, *Late North Caucasus culture*. The grave consisted of a deep burial (Grave 1b) covered and pit lined by stones and surrounded by a massive stone circle. Other human bones (Grave 1a) were found in the stone cover. This 45-55-year-old male from Grave 1b (KBD001) is a second-degree relative (grandfather or uncle) of the earliest dated individual yet identified with the genetic lactase persistence (LP) allele −13910*T [4], who was buried in Grave 2 of Kurgan 2. Dating: human bone, 2197-1975 calBCE (UGAMS-13455, 3690±30 BP)[4].

1.5 Komsomolec 1-Marfa
Country: Russian Federation
Region: Kirov district, Stavropol region
Coordinates: 43.977284°, 43.516373°
Excavation details: Excavation 'Nasledie', Stavropol and Eurasia-Department DAI, Berlin 2013-2015, license №2013-114, №2014-318, №2015-1036 (A. B. Belinskiy)

Summary:
The mound Komsomolec 1-Marfa is a single standing mound on the river terrace of Zolka creek, located 7.2 km west of the north-western outskirts of the city of Novopalovsk. Today the area is characterized by a piedmont steppe environment, but it was densely forested until the 19th century. The entire watershed is covered with former barrows, now mostly plowed out. Beside the mound, a circular ditch enclosure is visible on aerial and satellite imagery, as well as in a magnetometry survey conducted in 2009 and 2011 [11]. By the time of excavations, the mound's height was 8.5 m, and it had an oval shape with a diameter along the west-east axis of 62 m and 52 m along the north-south axis. The mound was built in several stages. Altogether, 60 graves have been excavated in the mound. Most of them are associated with the North Caucasus Culture (including Grave 37 from this study) and the contemporaneous Catacomb culture variant in the piedmont area. The initial mound dates to the later Maykop period, yet the central grave was destroyed. Beside the burials, a ditch with horse skeletons was opened, likely dating to Sarmatian times. Additionally, excavators discovered a ritual area with human skeletons beside the mound..

- **KMM016**, MARFA/K1/G37/I1, BZNK-946, Grave 37, individual 1. This grave is associated with the *North Caucasus Culture*. The burial consisted of a single inhumation of a male individual positioned on the back with knees bent upwards. The head was displaced by the later intrusion of catacomb 34. Dating: human bone, 2866-2574 calBCE (MAMS-28513, 4111±29BP; dating this study).

1.6 Krasnogvardeyskoe
Country: Russian Federation

Region: Krasnogvardeysk district, Stavropol region
Coordinates: 45.824999°, 41.583968°
Excavation details: Excavation 'Archaeological laboratory of the Stavropol State Pedagogical University', 1995

Summary:
The excavated barrow was a single standing mound situated 3 km to the east of the village of Krasnogvardeyskoe a terrace above the floodplain, 1 km south of the river Egorlyk [12]. The Egorlik is a steppe river linking the Stavropol highlands and the tributaries of the Manych river system. It occupied the top of a small natural promontory that intruded into the floodplain. The surface of the mound was overgrown; possibly, the mound had never been plowed. The mound was 2.97 m high and 60 m in diameter. Excavations revealed 23 burials, of which four are associated with the Yamnaya culture, including Grave 14 used in this study.

- **KNK003**, KRAS/K1/G14/I2, BZNK-1018, Kurgan 1, Grave 14, Individual 2. *Yamnaya*. Multiple-burial grave with three individuals (two adults and one child) in a rectangular stepped burial pit. Individual 1 was in a supine position on the back with the head facing east and knees bent. Bones were stained with ochre. Individual 2 (KNK003) was positioned next to Individual 1, also in the supine position with head facing east and bent knees, sprinkled with red ochre. Individual 3 was a poorly preserved young child near the arms of Individual 2. This child was not posed as the others, but the head did face east. The bottom of the pit was covered with brown wooden debris, and a chalky underlay was identified beneath it. The burial inventory included ceramic vessels, a piece of hematite, a piece of ochre, and three pebbles. Dating: human bone (from associated Individual 1 from Grave 14), 3012-2894 calBCE (MAMS-45960, 4327±26 BP; dating this study).

1.7 Kurganny 1
Country: Russian Federation
Region: Kirov district, Stavropol region
Coordinates: 44.022652°, 43.889008°
Excavation details: Excavation 'Nasledie', Stavropol 2017, license №2017-1775 (K. B. Kolesnichenko)

Summary:
The mound cemetery Kurganny 1 is located 1 km north of the settlement of Kurgan, near the northern outskirts of this village. Today the region is situated in a steppe environment, at the left terrace of the Kura River. The mound was 3.2 m high and 44 m in diameter. The kurgan embankment was constructed in two stages on top of Grave 17 (mound-shell 1) and Grave 16 (mound-shell 2). A total of 17 burials and two ritual complexes were opened inside the mound. The founding grave (Grave 17), included in this study, is most likely Eneolithic in date; others belong to the Steppe variant of Maykop culture (Grave 13), or date to the final Middle Bronze Age. One burial is Sarmatian in date, and the cultural affiliation of the other interments is ambiguous.

- **KUG002**, KUR1/K1/G13, Kurgan 1, Grave 13. *Late Steppe Maykop.* This burial included a single inhumation in a rectangular pit with rounded corners; the individual was buried in a sitting position, facing north-northwest. Dating: relatively dated to the last third of the 4th millennium BC, ca. 3300-3000 BCE.
- **KUG007**, KUR1/K1/G17, Kurgan 1, Grave 17. *Eneolithic Steppe culture*. This burial was the founding grave in the mound and consisted of a single inhumation in a rectangular pit with rounded corners, measuring 2.15x1.20 m, and oriented west-east. The individual was buried in a slightly crouched position on the back, with the head facing east. Below the skeleton was a thick layer of red ochre that was also visible on the entire skeleton. Dating: relatively dated to ca. 4300-4100 BCE.

1.8 Marinskaya 5
Country: Russian Federation
Region: Kirov district, Stavropol region
Coordinates: 43.905289°, 43.521881
Excavation details: Excavation 'Lomonosov Moscow State University', 'Institute of Archaeology RAS', 'Nasledie' 2007, license № 2007-687 (A. R. Kantorovich), 2007-840 (V. E. Maslov)

Summary:
The huge single-standing burial mound of Marinskaya 5 is situated on the high terrace of the river Kura. In 2009, it was excavated jointly by a team from Lomonosov Moscow State University, the Institute of Archaeology RAS, and the local heritage organization 'Nasledie'. The burial mound of Marinskaya 5 was slightly oval with a diameter of approximately 34-40 m and a height of 4.3 m. Four mound-shells, or construction phases, were discovered in the center of the mound. Three of these date to the Early Bronze Age Maykop era, while the last one was constructed later, during the Middle Bronze Age. Grave 33 was an above-ground burial vault surrounded by an oval ring of river pebbles. Directly on top of this grave, the first shell was constructed with earth and stone. Later, two Early Maykop burials were placed in the center, including burial 32, in this study. A second mound-shell followed. The third mound-shell was constructed after the interment of a late Maykop burial (Grave 25), in this study. Kantorovich, Maslov, and Petrenko have published the stratigraphy of these burials in Russian [13]. Paired cattle crania were found in many graves, including the grave associated with MK5004, in this study. These are some of the earliest documented cases of cattle symbolized as draft animals [14]. No additional graves were added to the site for approximately 600 years, when 18 graves were constructed, associated with the North Caucasus Culture, including Grave 13 from this study. Five Iron Age graves were added to the mound center, including MK5018 from Grave 1, studied here. The grave goods included in this burial are associated with the pre-Scythian period in the 10th to 8th century BCE.

The Marinskaya 5 complex was part of a comprehensive bioarchaeological study. Carbon and nitrogen stable isotopic data are discussed in Knipper et al. [7], arguing that the subsistence economy was likely pastoralist. The $\delta^{13}C$ and $\delta^{15}N$ values of the Maykop individuals are

generally higher than that of the following Middle Bronze Age cultures, suggesting a dietary shift away from grazing animals on C_4 plants or away from grazing in arid pastures [7]. However, these isotopic changes may also represent an environmental shift rather than a change in subsistence strategies. The mobility ranges of the Maykop individuals were likely limited to the piedmont steppe environmental zone. However, the carbon isotopic composition of bone collagen from some Marinskaya 5 cattle seems unusual for a piedmont steppe site as shown by high $\delta^{13}C$ values suggesting grazing on pastures with C_4 plants, which are not found in the area. This might indicate the importation of cattle from more steppe-like environments or herding mobility to these areas. The post-Bronze Age individuals are characterized by an isotopic composition with considerably higher $\delta^{13}C$ values, which may be a result of more C_4 plants in the diets of people and managed animals. Isotopic and botanical data from neighboring Iron Age sites confirmed millet cultivation and its direct consumption by Iron Age communities[7].

- **MK5004**, MAR5/K1/G25, BZNK-073, Kurgan 1, Grave 25. A 40-50-year-old male individual was buried in a disturbed *Late Maykop* inhumation within a square chamber surrounded by wooden posts and covered by wooden planks. A stone filling and packing was placed outside of the wooden chamber on top of the grave and the related mound embankment. The individual was placed in a flexed position on top of organic material. Below this was a plastered clay area ca. 2 m in diameter. Burial inventory included two bronze daggers, one golden earring, one ceramic vessel, and one bone arrowhead. Additionally, two cattle crania with nose rings, ovicaprid bones, and a badly preserved wooden object (possibly a yoke or fixing pole), were recovered outside the chamber. Dating: wood 3352-3094 calBCE (MAMS-13001/MA-110554 , 4498±30 BP) [14].
- **MK5005**, MAR5/K1/G32, BZNK-079, Kurgan 1, Grave 32. *Early Maykop* secondary inhumation of an adult female, aged 30-39, in a square pit with a ceiling constructed by wooden planks and organic matting, with a packing of mid-sized pebbles on top. The individual was placed in a flexed position on the left side, with the head oriented towards the east, with red ochre sprinkled on top of the skeleton. Although associated with the Early Maykop culture, this individual was genetically Steppe Maykop [4]. Dating: leather 3626-3377 calBCE (UGAMS-13047, 4720±25BP) [4]; human bone 3346-3096 calBCE (MAMS-11212, 4496±26BP) [4,13].
- **MK5017**, MAR5/K1/G13, BZNK-244, Kurgan 1, Grave 13. *North Caucasus culture*. Inhumation burial of an adult male, aged 20-35, in a deep-stepped, long rectangular burial pit with a stone frame on the step. The individual, placed within a wooden construction with a ceiling of two burned planks, was in a stretched supine position and oriented towards the southwest. The skeleton rested on top of white organic matter, grass remains, and red ochre. Burial inventory included eight bronze pendants (bracelets), bones of cattle, and the remains of a wooden pole or stick. Dating: animal bone 2865-2579 calBCE (MAMS-11216, 4120± 22 BP; dating this study).
- **MK5018**, MAR5/K1/G1, BZNK-100, Kurgan 1, Grave 1. Poorly preserved, *Pre-Scythian* inhumation of a female individual, aged 30-39, who was placed in a supine position with the head pointed towards the east. There was no noticeable pit. Grave

inventory included a ceramic vessel. Dating: tooth 901-813 calBCE (MAMS-51800, 2710 ± 18 BP; dating this study).

1.9 Nevinnomyssky 3

Country: Russian Federation
Region: Nevinomysk district, Stavropol region
Coordinates: 44.73486°, 41.938749°
Excavation details: Expedition 'Research Institute of Archaeology and Ancient History of the North Caucasus', Stavropol 2012, license №2012-1145 (S. V. Myachin)

Summary:
In 2012 several barrows were excavated in the mound field Nevinnomyssky 3. This group of at least 25 mounds is organized in a line running southwest to northeast along a ridge of a promontory at the right bank of the Kuban River at the confluence with the Bolshoi Zelenchuk, one of the largest rivers in the region[15]. A steppe environment characterizes this location, yet it is situated near the foothills of the Stavropol highlands. All mounds were relatively low and heavily plowed over. Mound 6 is located in the central part of the group beside the largest, Mound 1. It contained 14 interments, chiefly of Bronze Age date, and a stone circle of 15 m in diameter. It was built by communities associated with the Yamnaya culture, which started the mound with Graves 1 and 2. The earliest central grave (Grave 1) was not preserved, yet a stone stela was found at its location. The first surviving Yamnaya grave was Grave 2. Two graves (10 and 13) are associated with the North Caucasus Culture; Grave 14 was of Catacomb (Manych type) affiliation.

The largest group of five burials (Graves 5, 6, 7, 8, and 12), including Grave 5 from this study, were post-Catacomb or Lola type burials with similar characteristics. The second individual in this study was recovered from Mound 7. This mound was surrounded by a ditch and revealed the remains of at least two mound-shells as well as a stone circle. Excavators discovered a complex sequence of burials in pits and catacombs in this mound. A total of 12 burials were excavated from an area of 16 m^2. Grave 23, included in this study, was the central grave and dates to the Maykop epoch. It was built over a sequence of two Yamnaya burials, a North Caucasus Culture burial, two Catacomb (Manych type) burials, and a post-Catacomb burial, the last belonging to the Lola group of monuments.

- **NV3001**, NEV3/K6/G5, BZNK-312, Kurgan 6, Grave 5. *Lola/post-Catacomb* single inhumation in a burial pit (reduced catacomb outside of the earlier stone circle). A male skeleton was positioned on top of organic bedding, on the left side of the body, with the head facing east-southeast. Anthropological examination revealed several healed fractures of the hands and ribs and severe and chronic inflammation in parts of the upper body and the skull [16]. The musculature of the upper body was well-developed, indicating additional stress on the upper extremities. More pronounced muscle development in the left arm, and a fracture in the left hand, characterize the individual as left-handed. The bones of the right leg likewise show the consequences of a traumatic injury resulting in ossification of the femur after inflammation. The

individual likely suffered from myositis, an inflammation of the skeletal musculature, which can be caused by infections, instabilities of the immune system, or acute toxicity. An elevated level of trace elements such as arsenic, lead, and mercury, were found on the hands, most likely associated with metallurgical activities. The burial equipment includes a crucible and a tuyere made from clay, a whetstone, and a cattle scapula. This individual was a part of a genomic study that showed a genetic ancestry consistent with the Lola culture [4]. Dating: human bone 2127-1924 calBCE (MAMS-29812, 3631±22BP) [4].

- **NV3003,** NEV3/K7/G23, BZNK-1003, Kurgan 7, Grave 23. This *Early Maykop* burial consisted of a single inhumation in the remains of a rectangular burial pit. The male individual was in a crouched position with the head facing north. The individual was placed on top of dark-brown organic matter. Chalk was placed at the knees and below the skull, while red ochre was placed below the pelvis. Dating: human bone 3776-3652 calBCE (MAMS-42353, 4953±20BP; dating this study).

1.10 Progress 2

Country: Russian Federation
Region: Kirov district, Stavropol region
Coordinates: 43.822691°, 43.350278°
Excavation details: Excavation 'Nasledie', Stavropol 2009-10, license №209-545 (S. Ja. Berezin)

Summary:
Progress 2 is located in the piedmont steppe zone of the North Caucasus, 20 km from the archaeological sites of Marinskaya 5 and the Komsomolec 1-Marfa mound. Five of the ten mounds of the group were excavated prior to gravel mining from the former bed of the river Malka [17,18]. The river terraces here are dotted with burial mounds, of which several were excavated in the 1970s. Most burials in Progress 2 are affiliated with the North Caucasus Culture, but Mound 1 and 4 were initially built on top of Eneolithic inhumations. Two of these Eneolithic graves were sampled and produced genome-wide data [4]. Mound 1, Grave 37 (PG2001), included in this study, is the founding grave. This individual dates to the Eneolithic. Isotope analysis of this individual's bone collagen showed $\delta^{13}C$ and $\delta^{15}N$ values similar to that of individuals from sites in the same environmental zone, such as Marinskaya 3 and 5 [7], suggesting a pastoralist lifestyle. These carbon and nitrogen isotopic values were high compared to other Eneolithic individuals associated with the Darkveti-Meshoko group [3], who are regarded as being members of the first sedentary agro-pastoralist community in the North Caucasus mountains based on the recovery of settlement architecture and domesticated animals and plants. Two additional burials in Mound 1 are associated with the Yamnaya culture, while 11 graves include material culture associated with the North Caucasus Culture, including one individual included in this study (PG2002). Mound 4 contained two Eneolithic founding graves (9 and 12), including the individual from Grave 12 (PG2003), which is included in this study.

- **PG2001**, PROG2/K1/G37, BZNK-113, Kurgan 1, Grave 37. *Eneolithic Steppe.* This grave included a single inhumation of an adult male, age 26-49, in an oval grave-pit. The upper part of the body was stretched supine, legs in a crouched position on the left side, and the head was positioned towards the east-northeast. Ochre was sprinkled on top of the entire body. Burial inventory included a long flint blade, a flint adze, a flint projectile head and another flint object. . Dating: human bone 4994-4802 calBCE (MAMS-13011/ MA-110564, 6012±28). Charcoal 4338-4074 calBCE (MAMS-13010/ MA-110562, 5397±28 BP), indicating radiocarbon offset from the diet [4].
- **PG2002**, PROG2/K1/G25b, BZNK-303, Kurgan 1, Grave 25B. *North Caucasus culture*. Double inhumation in a rectangular stepped grave-pit. Skeleton 25A was positioned in the southwestern corner of the pit, in a stretched supine position, with the head pointed towards the southeast (though the skull exhibited extensive fragmentation). The skeleton rested on top of dark-brown organic material. Skeleton 25B (PG2002), a 35-45-year-old female, was placed in the pit center over the covering of skeleton 25A. The skeleton of 25B was placed in a stretched supine position, head towards the northwest, on top of dark-brown organic material, and with ochre at the feet. Burial inventory included animal bones on the stone steps of burial 25A and ovicaprid bones at the lower legs of 25B. Additionally, skeleton 25B was adorned with agate, bone, and possible white metal beads on the neck and agate and bronze beads at both wrists (bracelets). Dating: human bone 2476-2303 calBCE (MAMS-29815: 3929±22 BP) [4].
- **PG2003**, PROG2/K4/G12, BZNK-061, Kurgan 4, Grave 12. *Eneolithic Steppe.* Single inhumation of an adult male, aged 25-29, in a rectangular grave-pit with rounded corners. The torso was supine, legs were leaning to the right side, and the head was facing towards the east. The skeleton was thickly packed in red ochre and had undergone a complex trepanation [18]. Dating: human bone 4240-4047 calBCE (MAMS-11211, 5305±25 BP)[18].

1.11 Rasshevatsky 1

Country: Russian Federation
Region: Novoaleksandrov district, Stavropol region
Coordinates: 45.537249°, 41.116544°
Excavation details: Excavation 'Nasledie', Stavropol 1998-2000, license №2000-252 (V. L. Rostunov)

Summary:
The site of Rasshevatskiy 1 is part of a series of barrow groups running in a line from west to east. The vegetation today is an herb-steppe with larger patches of forest-steppe vegetation [19]. The Rasshevastsky 1 cemetery includes 27 mounds spread across 2 km. Mound 21, excavated in 2000, was 110m long and 85m wide. It was built in five construction layers (mound-shells) of different forms and earth compositions. Mound-shell 2 was built over the empty burial pit 3 in the Yamnaya period; Graves 9, 11, and 13 (which was included in this study) were placed within the shell. These graves were also associated with the Yamnaya epoch. Two individuals from Mound-shell 2 were included in previous paleogenomic studies [4], and Grave

11 revealed the oldest evidence of an early form of *Yersinia pestis* [20]. The complexes of Mound 21 from Rasshevatsky are part of a larger bioarchaeological study, scheduled for publication in 2022/23.

- **RK1002**, RAS1/K21/G13, BZNK-035, Kurgan 21, Grave 13. The burial consisted of a single inhumation of a 35-45-year-old *Yamnaya* female in a rectangular pit. The skeleton was in a supine position, legs crouched, and head oriented towards the northeast. The body was placed on top of dark organic material, chalk, ochre, and charcoal. The individual produced genome-wide data, and ancestry was consistent with the Yamnaya culture. Burial inventory included a ceramic vessel that was placed beside the body. Dating: human bone 3331-3013 calBCE (MAMS-29818, 4447±22 BP) [7].

1.12 Rasshevatsky 4
Country: Russian Federation
Region: Novoaleksandrov district, Stavropol region
Coordinates: 45.535978°, 41.059517°
Excavation details: Excavation 'Nasledie', Stavropol 1999, license № 1999-681 (Ya. B. Berezin)

Summary:
Rasshevatsky 4 is a line of mounds running west to east, parallel with the mounds of Rasshevatsky 1, which is located only 4 km northeast. Six mounds were excavated during rescue excavations in 1999. The most eastern was Mound 1 with an oval form, a dimension of 18-35 m, and a preserved height of 0.36 m. This mound included 3 Catacomb burials, including Grave 5 from this study, as well as 2 burial pits dating to the post-Catacomb culture. Two individuals from Mound 1, including RK4002 from this study, produced genome-wide data [4], which confirmed a genetic ancestry consistent with the Catacomb culture.

- **RK4002**, RAS4/K1/G5, BZNK-304, Kurgan 1, Grave 5. This *Early Catacomb* burial consisted of a single inhumation of a male skeleton in a T-shaped catacomb. The skeleton was left on top of organic material, in a supine position with knees bent upwards, and the head was positioned towards the north. Dating: human bone 2662-2474 calBCE (MAMS-29820, 4050±22 BP) [4].

1.13 Sharakhalsun 6
Country: Russian Federation
Region: Turkmen district, Stavropol region
Coordinates: 45.725651°, 43.239869°
Excavation details: Excavation 'Nasledie', Stavropol 2001, license №2001-791 (A. V. Yakovloev)

Summary:

The Sharakhalsun archaeological sites are a part of a large, dense cluster of several hundred burial mounds, located approximately 70 km south of Elista, primarily organized in a line from west to east. The cemetery, situated along the bank of the Kalaus river near the Maynch water reserve, has been excavated by several 'Nasledie' field campaigns and through rescue excavations, which resulted in the recovery of over 330 graves. Mound 2, included in this study, was excavated in 2001. It was 50 m in diameter, 3 m in height, and contained four complexes from different cultural formations. These complexes contained the remains of wooden wagons, including the oldest one to date (Grave 18) [21]. Grave 18 was part of a Maykop cluster dating to the second half of the 4th millennium BC, and it exhibited influences from Maykop and Yamnaya traditions. The individual in Grave 18 (SA6004) had suffered and survived injuries consistent with an accident involving a wagon and/or draft animals [22]. This individual was previously analyzed using paleogenomics techniques [4], and the remaining Mound 2 burials are part of an ongoing bioarchaeological study. Mound 5 was situated in the eastern part of the cemetery and had a diameter of 36 m and a height of ca. 2 m. It included 16 burials, of which Grave 7, studied here, was the initial one. The other graves are associated with the Yamnaya, Early and Late Catacomb, and the Iron Age cultures.

The individuals buried in the Sharakhalsun 6 kurgans represent communities in the dry Caspian steppe reliant on a pastoralist diet (i.e., meat and other animal-derived products for protein). The mobility ranges of the herds likely did not extend to the more moist herb-grass areas of the piedmont steppe zone [7]. Furthermore, cattle herding strategies may have differed from ovicaprids, which were pastured at drier locations. A fish component of the diet is possible from the considerably high stable nitrogen isotope values [7]. Steppe rivers have been a part of the migration routes of Caspian sturgeon before modern regulations.

- **SA6004**, SHAR6/K2/G18, BZNK-003, Kurgan 2, Grave 18, Late Steppe Maykop. This exceptional grave is an inhumation of a 26-49-year-old male sitting on a four-wheeled wooden wagon in a narrow catacomb. No additional grave offerings were found. The biological anthropological examination of this individual revealed many pre- and perimortem injuries [22]. Dating: wood, 3359-3034 calBCE (GIN-12401, 4500±40 BP) [4,14,23]. Note: In Reinhold et al. [14], the site and mound number for SA6004 were transposed in the text but are correct on the photographs. The correct label of this site is Sharakhalsun 6, Mound 2.
- **SA6014,** SHAR6/K5/G7/I1, BZNK-1000), Kurgan 5, Grave 7, individual 1. *Late Steppe Maykop*. The burial consisted of two inhumations in a catacomb. Both skeletons were found in a flexed position on the left side, with the heads facing south, placed close to each other. One individual (SA6014) was a 30-39-year-old female, while the second individual was a child. Red ochre and white organic material were found below the bodies. A ceramic vessel was placed near the feet. At the head of the adult, a fragment of wood and several dark gray, green-grey, and white shell beads were found. Dating: human bone, 3366-3029 calBCE (GIN-13205, 4510±50 BP) [24].

1.14 Tipki 1

Country: Russian Federation
Region: Apanasenkovo district, Stavropol region
Coordinates: 45.737683°, 44.072967°
Excavation details: Excavation 'Nasledie', Stavropol 1997, license № 1997-741 (S. V. Lyakhov)

Summary:
Four mounds form the barrow group of Tipki 1. It is located on a gentle slope on the left bank of the Kalaus river, 26.5 km northeast of the village Raguli. The Kalaus river is an important transitional route between the Caspian steppe and the North Caucasus piedmont zone.
Mound 2 had a diameter of 62.0 m and a height of 2.86-2.48 m prior to excavations. The construction revealed three building phases, i.e., mound-shells. A ditch surrounded the central part of the mound, and a ritual complex with three concentrations of animal bones was found in the northeastern part of the mound. A total of 17 tombs were identified in the mound, of which tombs 15 and 16 are the founding graves and belong to the North Caucasus Culture. Graves 4-14 date to different periods of the Bronze Age. Whereas Graves 7, 10, 11, and 12 are associated with mound-shell 2, Graves 13 and 14 are associated with mound-shell 3. Graves 8 and 9 also appear to be associated with mound-shell 3. Graves 1 and 17 date to the Early Iron Age, and Grave 3 dates to the Middle Ages.

- **TIP001**, TIP1/K2/G4, BZNK-1024, Kurgan 2, Grave 4, *Lola/post-Catacomb culture*. This burial consisted of a single inhumation of a male individual in a rectangular pit with rounded corners. The skeleton was left in a crouched position on the left side, with the head oriented to the north. A typical Lola bone pendant or belt hook was found with the human remains. Dating: human bone, 2192-1887 calBCE (GIN-10311, 3640±50 BP) [25].

1.15 Vinsady 4
Country: Russian Federation
Region: Predgorny district, Stavropol region
Coordinates: 44.061002°, 42.965117°
Excavation details: Excavation 'Nasledie', Stavropol 2014, license № 2014-193 (A. V. Lychagin)

Summary:
The site of Vinsady 4 is part of a broader cluster of mounds at the western outskirts of the city Pyatigorsk. The barrow field consists of at least 12-13 mounds, of which 9 were recently excavated but given different site names, including: Vinsadsky 2, Vinsady 4, Vinsady 5, and Skachki 2. More mounds might have been situated beneath a modern industrial park and former military base. Vinsady 4 Mound 2 was situated on the southern slope of a low ridge descending from northwest to southeast in the direction of the Podkumok River valley. The mound was circular in plan, with a diameter of ca. 28 m and a preserved height of ca. 1 m. Inside, the stone cover of the initial mound-shell was found surrounding the Maykop founding Grave 11. Fifteen more graves were dug into this mound, most of them associated

with the North Caucasus Culture or the Late Bronze Age. The seven North Caucasus burials (Graves 1-5, 8, 12-13) were arranged in two circles surrounding the central grave. Graves 5 and 8 formed the second outer circle.

- VS2001, VIN4/K2/G8, BZNK-1008, Kurgan 2, Grave 8. *North Caucasus culture.* Grave 8 is an unconventional, poorly preserved wooden chamber. The body parts of at least two individuals were disarticulated and placed in the grave. In the center, a skull was placed on an arm with a shoulder blade, long bones, and hand in anatomical position. Another set of hand bones of similar size were found. Both were placed in an area of red ochre. Somewhat apart from the skull, a third hand was found, belonging to a much larger individual. No burial inventory was found. Dating: human bone, 2866-2578 calBCE (MAMS-45971, 4120±27 BP; dating this study).

1.16 Zolotarevka 1
Country: Russian Federation
Region: Ipatovo district, Stavropol region
Coordinates: 45.664363°, 42.588243°
Excavation details: Excavation 'Nasledie', Stavropol 2000, license №2000-400 (A. A. Kamykov)

Summary:
The Zolotarevka mound groups represent several distinct clusters of barrows in the vicinity of the village Zolotrarevka at the watershed of the rivers Kalaus and Bolshaya Kugulta, c. 80 km upstream of Krasnogvardeyskoe. The area is a wide plain shaped by the small meandering river. All mounds have been heavily plowed over and were relatively low prior to excavation. The Zolotarevka 1 group is formed by a line of at least 79 mounds, running southeast to northwest. The excavated part was situated at its eastern end. The excavations could reveal a chronological shift of the mounds starting from the Maykop period. Mounds 23 and 24, which contained the individuals in this study, were two small constructions south of the major line. Each had a single grave.

- **ZO1005**, ZO1/K23/G1, BZNK-1030, Kurgan 23, Grave 1. *Lola/post-Catacomb culture*. Burial consisted of a single inhumation in a catacomb with a broad, rectangular entrance pit; The female individual was placed in a crouched position on the left side, with the head facing east. Many beads made from faience, ceramic, antimony, and gemstones were placed at the head and torso. A metal object resembling a perforator was also placed near the head. Other burial content includes ovicaprid bones, a flat thin stone disc, an animal tooth pendant, and a stone pestle placed near the feet. Dating: bone, 1953-1700 calBCE (GIN-12407, 3520±40 BP; this study)
- **ZO1006,** ZOL1/K24/G1, BZNK-1031, Kurgan 24, Grave 1. *Lola/post-Catacomb culture,* single inhumation of a male individual in a catacomb with a broad, rectangular entrance shaft. The individual was placed in a crouched position on the left side with the head facing east, with a slight twist to the south. A ceramic vessel

was placed at the arms, and ovicaprid bones were placed at the hands. Bones belonging to adult cattle were also placed in the grave. Dating: human bone, 2271-2038 calBCE (MAMS-42370, 3740±21 BP; dating this study).

1.17 Zolotarevka 2

Country: Russian Federation
Region: Ipatovo district, Stavropol region
Coordinates: 45.658644°, 42.605994°
Excavation details: Excavation 'Nasledie', Stavropol 2000, license №2000-400 (A. A. Kamykov)

Summary:
Zolotarevka 2 is one of several mound cemeteries situated at the watershed of the Kalaus and Bolshaya Kugulta rivers. It forms a cluster of mounds that continues the Zolotarevka 1 line of barrows to the southeast. Today it is situated in a grass steppe environment. The second group consisted of four mounds that were leveled entirely by plowing prior to excavation. All mound excavations occurred in 2000 during rescue excavations. The fourth mound held three Yamnaya graves, including Grave 4, which contained the remains of an individual from this study.

- **ZO2002**, ZOL2/K4/G4/I2, BZNK-281, Kurgan 4, Grave 4, Individual 2. This *Yamnaya* burial consisted of 4 individuals placed in two couples within a large shaft. The individuals were placed in a typical Yamnaya pose. Three males and one 40-55-year-old female (ZO2002) were buried together. Bronze and bone beads were found with ZO2002. Other burial inventory included a silex flake, four pieces of red ochre, and chalk. Indeterminate faunal remains were also present. Dating: human tooth, 2850-2501 calBCE (MAMS-50904, 4089±22 BP; dating this study); human bone, 2843-2492 calBCE (MAMS-51322, 4069±22 BP; dating this study).

2. Archaeological Site and Sample Information for South Caucasus

2.1 Alkhantepe

Country: Azerbaijan
Region: Jalilabad district
Coordinates: 39.360117°, 48.462000°
Excavation details: Excavation Murgan Neolithic-Eneolithic expedition, 2008-2010, 2012 (T. I. Akhundov)

Summary:
The site of Alkhantepe was excavated by Tufan Axundov [26] and is located in Cəlilabad (Jalilabad) Rayon, in SE Azerbaijan, a region characterized by higher annual precipitation compared to the rest of the country [27]. The site is located 4 km north of Uçtepe village. Today the site is about 60 km from the shore of the Caspian Sea, but paleolandscape studies indicate that the Caspian coastline extended closer to the site in the past [28]. Furthermore, palynological

research at the site revealed an environment of lowland broadleaved forest and, to a lesser extent, Eldar pine [29]. During the ca. 200 years of occupation, a qualitative change in the grass vegetation was observed. The site has a flat morphology and covers an area of at least 4 hectares. Cultural layers extend 3 m beneath the modern surface. In an excavated area of 10 x 20 m, a sequence of seven building horizons have been recorded; some can be divided into sub-horizons. They are formed of various buildings, including circular and rectangular constructions, circular and rectangular pit-houses with mudbrick or pisé walls, as well as above-ground brick and wattle and daub structures. The settlement of Alkhantepe represents the late stage of the Leilatepe cultural tradition and can be dated to the 4th millennium BC. In the settlement layers, the remains of 13 burials of different age groups were found in various levels and areas. Burials of infants were chiefly found inside large clay vessels, some in specific burial pits. They are oriented with the head mainly to the southwest. Of the adults, one individual from Burial 12 was investigated in this study.

The Neolithic economy of Alkhantepe was based on farming, including agriculture and small-scale herding of sheep, goats, and cattle. Cattle figurines are also present at the site. Alkhantepe is one of the earliest locations in the South Caucasus where metallurgical production using copper and lead is attested [26,27].

- **ALX002**, Alkhantepe burial N2, AZ12, Burial 12, *Chalcolithic Leilatepe tradition*. Male. This poorly preserved burial included the remains of a male individual in a shallow pit. The skeleton was placed in a crouched position, on his side, with the head facing northeast. The burial inventory included a lead ring made of wire placed on top of the lower leg. This individual was a part of a paleogenetic study and produced genome-wide results [30]. Dating: human tooth, 3776-3651 cal BCE (MAMS-40330, 4950±23 BP)[30].

2.2 Göytepe
Country: Azerbaijan
*Region:*Tovuz District
Coordinates: 40.969958°, 45.705183°
Excavation details: Tovuz Archaeological Expedition of the Institute of Archaeology and Ethnography, Azerbaijan National Academy of Sciences 2015 [31].

Summary:
The archaeological investigations at Göytepe, a large Neolithic mound of the Shomutepe archaeological culture in the western Republic of Azerbaijan (Tovuz district), have been in progress since 2008. Göytepe is situated approximately 10 km east of Tovuz in the middle of the Kura Valley. Located at the western edge of the Zayam River delta, one of the tributaries of the Kura River, the site consists of a circular mound with a diameter of 145 m and a height of 9 m. The surrounding region is known as the Qazakh-Ganja plain. The first excavations started in 2008 with the Azerbaijan-Japan mission [32].

During the 2015 field season, two graves dating to the Late Bronze Age were found in the upper layer of the Göytepe Neolithic settlement. One of the graves (conditionally marked as "Grave No. 1") was encountered at the beginning of the 4th building level in courtyard No. 11, square 1AI.

A bone sample was taken from the skeleton (probable female) in Grave No.1 and analyzed at the radiocarbon laboratory of the Institute of Accelerator Analysis/AMS in Tokyo, Japan (IAAA-161182) [31,33]. Later, a tooth from the same individual was radiocarbon dated again at the Curt-Engelhorn-Centre for Archaeometry (MAMS) in Mannheim, Germany (MAMS-40332). Both analyses yielded consistent dates, placing the burial at the end of the Late Bronze Age and the beginning of the Iron Age.

The grave is associated with the Khojaly-Gadabay archaeological culture (14th-9th centuries BCE) [34,35] of the Late Bronze Age to Early Iron Age based on mortuary artifacts. The Khojaly-Gadabay culture covered almost the entire territory of the modern Republic of Azerbaijan but exhibited local variation. The communities practiced farming and pastoralism. The culture is characterized by settlements with defensive walls, cyclopean buildings, and different funeral practices (graves, kurgans, stone cysts/boxes). Settlements were located mainly in the foothills and uplands of the Lesser Caucasus. Funeral equipment is replete with ceramics, bronze, and bimetal products, and burials also contain the remains of domesticated horses. The period of this culture is characterized as coinciding with a demographic expansion. The origins of the culture remain debated, with suggestions ranging from Proto-Cimmerians to affiliations with Late Bronze-Early Iron Age cultures of northeastern Anatolia and northwestern Iran.

- **GYT001**, Grave No. 1, goy#1x, Courtyard 11, Square 1AI. *Khojaly-Gadabay culture*. Probable female. Grave goods indicate an association with the Khojaly-Gadabay culture, Late Bronze Age to Early Iron Age. Dating: human tooth, 1014-906 calBCE (MAMS-40332, 2812 ± 20 BP; dating this study); human bone, 1055-916 calBCE (MAMS-36860, 2836 ± 21 BP; dating this study); human bone, 1122-974 calBCE (IAAA-161182, 2872 ± 24 BP, δ13C -17.95 ± 0.36 %).[31,33]

2.3 Qabala

Country: Azerbaijan
Region: Qabala District, Shaki-Zagatala Economic Region
Coordinates: 40.8900°, 47.7082°
Excavation details: Qabala in Antique and Early Middle Ages Expedition (since 2011) (Jeyhun Eminli)

Summary:
The Salbir (Chukhur Qabala village) and Uzuntala (Soltannukha village) sites are located in the Qabala (Qəbələ, Gabala) district of Azerbaijan, as part of an ancient city situated along historical trade routes. Qabala district is characterized by riverine-cut, mountainous terrain over about half the area of the region, with several strategic mountain passes. The original city, the fortified capital of the ancient kingdom of Caucasian Albania, was developed from

the end of the 4th century BCE onwards and represents early state formation in the South Caucasus during the fall of the Achaemenid Empire [36]. The subsequent kingdom of Caucasian Albania comprised a territory to the Sulak River in Darband in the north and south to the Araxes (Aras) River, while ranging from Caucasian Iberia (centered on present-day Eastern Georgia) in the west to the Caspian Sea in the East. Caucasian Albania and its cities, including Qabala, were known to Roman scholars Pliny the Elder and Ptolemy, having repeated contacts with Roman armies [36].

In the 5th century CE, Caucasian Albania became a province of the Sasanian Empire, which moved the political center of the province from Qabala to Bardha'a. Despite this move of the local seat of power, Qabala maintained its importance as a cultural and economic center for the wider region [36]. Historical confusion surrounding the founding of the city by the Sasanian Empire is best explained by a large construction project in the 5th and 6th centuries to fortify the city's defensive walls [36].

The city contains three main areas. Chaqqali covers about 50 hectares and is located in the south-east of Chukhur Qabala village between the Garachay and Gochalan rivers. This area appears to have been strongly fortified having been enclosed by natural canyons to the east, west, and north sides, while a rampart was used as a defensive wall to the south. The occupation of Chaqqali roughly spans the Antique period in Azerbaijan (4th c. BCE - 1st c. CE), which was built on top of remains dating to the Bronze and Iron Ages. Archaeological research indicates that Chaqqali was built in the late 4th c. BCE and remained in use until the 1st c. CE [36]. This area contains administrative buildings of various shapes and sizes. Chaqqali began to decline at the end of the 1st c. CE, while settlement of Qabala continued into the Medieval period in the areas of Salbir and Qala.

Salbir and Qala are located on high plateaus, with walls on the north, south, and west sides, while a cliff gave natural defense to the east, enclosing an area of about 25 hectares. An occupational layer dating to the late Antique period (2nd-3rd c. CE) represents the earliest cultural horizon. Occupation of Salbir declined in the 11th c. CE, while Qala continued to be used into the 18th c. CE.

The Salbir area has three main occupational phases: 1st-3rd c. CE, 5th-6th c. CE, and 8th-11th c. CE. The Late Antique phase was shown in relatively thin archaeological layers, which contained floors with unbaked bricks and postholes, ash deposits, and ceramic fragments. Throughout the archaeological deposits of Salbir, graves were recovered that date from the late Antique period to the 15th c. CE, which included graves of simple ground pits, catacombs, jar burials, and tile-lined cists. Catacomb burials are thought to be related to Sarmato-Alan groups from the north.

In the territory of the Qabala region, burials related to 1st c. BCE-3rd c. CE were investigated. The single graves and cemeteries were studied in Chukhur Qabala village (Salbir site), Soltannuhka village (Uzuntala site) and Gushlar village [37–39].

A comprehensive understanding of subsistence strategies at Qabala based on the remains of plants and animals has yet to emerge due to a lack of systematic examination of these materials. Of the animal bones recovered from occupational and mortuary deposits at the site, sheep, goats, and pigs are represented in greater numbers than cattle, horses, and camels [40]. While relative abundances for skeletal faunal remains from Qabala are unavailable, zooarchaeological research at Mingachevir, a contemporaneous city located about 75km to the southwest of Qabala, provides some insight into the animal management economies in ancient Caucasian Albania. From archaeological deposits dating to the 3rd-8th c. CE at Mingachevir, the percentages of identified faunal skeletal remains were as follows: sheep and goat - 29.54%; cattle - 25%; pig 22.72%; horse 9.09%; wild game (deer, wild boar, etc.) - 7.5%; birds (domestic and wild) - 5.3%; and dog - 0.75% [41]. These findings are reported to broadly reflect the pattern of skeletal faunal remains from Qabala [40]. Dairy production is evidenced at Qabala and other ancient cities throughout Azerbaijan through the recovery of wide-mouthed ceramic milking vessels and ceramic churns, and wool production is indicated by iron shears [40]. Agricultural production at these ancient cities largely focused on wheat, barley, and millet, and iron sickles have been found throughout the archaeological deposits [42]. Macrobotanical remains also include grape pips, and wine production is evidenced by ceramic household jugs containing wine residue and grape pips, stone troughs for mashing grapes, and cisterns for fermentation and storage, which have been found at Qabala [40].

- **QAB001**, QQ 1 SK1, burial 1, skeleton 1. 26-28-year-old male. Pair burial excavated in 2013, at the Salbir site. The grave chamber with a depth of 100 cm, measuring 130 cm x 230 cm, located 135 cm below the surface of the earth was dug into the geological clay layer. The chamber is oriented northeast – southwest. Two human skeletons were uncovered in the central part of the tomb – a male and a female (see below). The heads of both skeletons are located in the northeast, and their legs in the southwest. The female is positioned on the right side of the body, with legs bent at the knee, arms bent at the elbow, and hands under the head. The male is positioned on the left side of the body, legs bent at the knee, and left arm bent at the elbow. The right arm of the male extends towards the female, with a hand on the face of the female. The two individuals had significant dental calculus accretions. The grave is accompanied by an inventory of grave goods, as well as an iron sword, bronze mirror, various beads, glass goblet, and ceramic vessels. Dating: human bone, 81-227 calCE (MAMS-36851, 1887±24 BP dating this study).
- **QAB002**, QQ1 SK 2, burial 1, skeleton 2. 18-20-year-old female (see above). Dating: human bone, 76-210 calCE (MAMS-36852, 1905±19 BP; dating this study).
- **QAB005**, QQ 10 SK1, burial 10, skeleton 1. Soltannukha 2014, Qəbir No.1 (Grave No.1) [38]. Jar burial containing two human skeletons [38]. The burial was detected at a depth of 90cm. The sequence of the burials can be observed with the relative positioning of the skulls and post-cranial bones placed in the vessel. The skulls are to the north-west and placed face to face. Zoroastrian burial practices are possibly observed in the secondary burial of the human remains in the jar. The burial inventory includes ceramic vessels, bronze adornments, and various beads. Skeleton 1 (QAB005) was a juvenile approximately 14 years old. Skeleton 2 (not sampled) was

an elderly female over 50 years of age. Dating: human bone, 360-172 BCE (MAMS-36855, 2191±21 BP; dating this study).

2.4 Qızqala

Country: Azerbaijan
Region: Şərur District of the Autonomous Republic of Naxçıvan
Coordinates: 40.3661°, 49.8372°
Excavation details: Naxçıvan Archaeological Project survey and excavations 2013-2016, University of Pennsylvania (L. Ristvet), Emory University (H. Gopnik), and University of Chicago (E. Hammer).

Summary:
Excavations of Qızqala 1 occurred between 2014 and 2016, overseen by Veli Bakhshaliyev from the Azerbaijan National Academy of Sciences and Hilary Gopnik from Monash University, Australia. The excavated site consists of both a settlement complex and a cemetery dating from the Middle Bronze Age through the Early Iron Age [43,44]. These are located topographically below an approximately 2 ha hilltop fort with occupation in the same periods [45]. Due to the heavy erosional deposits around Qızqala over the MBA levels, the exact extent of the settlement cannot presently be determined. However, a survey identified dense MBA pottery scatters that span an area of approximately 8–10 ha [45].

The landscape around Qızqala hosts an expansive area for funerary practice, suggesting a large community occupied this area. The cemetery space occupies roughly 100 ha on the hilltops and in the valleys north of the settlement and contains at least 131 identifiable kurgans, of which 11 burials were systematically excavated [46,47]. MBA funerary activity also extends to the eastern bank of the Arpa River, where excavations at Yaycī identified further MBA kurgans. The kurgans at Qızqala are large, mounded pit burials encircled with stone cromlechs, and they are positioned along hill ridges or clustered in valleys. These burials were prepared for 1 to 3 individuals and contained abundant faunal remains [48] and objects, including painted and incised ceramics, lithics, weaponry, and jewelry [46,49]. Isotopic studies of individuals from these kurgans suggested some local population mobility, probably for herding purposes [47]. The poor preservation of remains from the kurgan burials did not permit sampling calculus. Rescue excavations took place at the southeast of the site, where roadwork excavations exposed a further extent of the MBA Qızqala cemetery. Excavations focused on lifting exposed human remains but did not investigate burials in depth. Burials in this area appeared to be pits or rock-lined pits without evident objects. Pits were cut into levels with fragments of black painted red pottery, characteristic of Middle Bronze Age ceramic traditions. Individuals from these burials had preserved calculus and were selected for sampling for this study.

The Middle Bronze and Early Iron Age inhabitants of Qızqala engaged in agropastoralism that included seasonal crops and herding mobility. Faunal assemblages are dominated by ovicaprid remains, with some cattle and equids as well [48].

- **QZQ002**, qq15 burial 10 LRM33, *Middle Bronze Age*. This individual was an adult male aged 30-35, buried in a flexed position oriented N-S with head in the north, facing east. Burial was a pit with possible rock lining with no evident mounding or burial accompaniments. Dating: relative ca. 2300-1500 BCE
- **QZQ003**, qq15 burial 114 LLI1, *Middle Bronze Age*. This individual was a juvenile aged 11-13, buried in a flexed position oriented N-S with head in the north, facing west. The burial appeared to be a pit burial with no evident mounding or burial accompaniments. Dating: relative ca. 2300-1500 BCE

2.5 Mentesh Tepe
Country: Azerbaijan
Region: Tovuz district
Coordinates: 41.006711°, 45.660667°
Excavation details: Excavation 'CRNS, UMR 7192' 2011-2014 (B. Lyonnet, F. Guliyev)

Summary:
Mentesh Tepe is a small settlement mound in the middle Kura valley. It was excavated in 2008 by a French team, partly in the framework of a joint German-French project focusing on the Neolithic in the Kura valley. The site revealed settlement layers of the 6th millennium BCE (Period I, Neolithic), the 5th (Period II-III, Chalcolithic), and two kurgan burial mounds (Period IV, Early Bronze Age), one dated to the Early Kura-Araxes period (late 4th/early 3rd millennium BCE), and the other dated to the Early Kurgan period (ca. middle of the 3rd millennium BCE).

The collective Neolithic Grave 342 was excavated in 2011-12 by a team led by Bertille Lyonnet [50], and one individual from this grave was included in this study (MTT001). The pit was found beside circular buildings but cut into the settlement layers. It revealed the remains of 31 individuals dating to the early 6th millennium BCE. The individuals might, however, be affected by freshwater reservoir effects. The circular pit had a diameter of approximately 3.8 m, with a dome-shaped construction of mud bricks that was partly filled before the burials, which contained the remains of 31 individuals. Some of them were placed in crouched positions alongside the pit walls, sometimes in pairs. Others were thrown into the center of the pit irregularly. Eleven individuals were adults, 20 were subadults, and no children under one year were present. Sex determination was possible for 12 individuals, among them eight females, four males. The health status of the individuals was rather good. Dental calculus was present in low quantities. Adornments made of bone and fish bone, obsidian blades, and a pottery fragment have been found among the skeletons. Neolithic Mentesh Tepe had an economy based on domesticated animals and agriculture. Domesticated ovicaprids dominate the faunal assemblages, and smaller numbers of cattle, pig, and dog remains have also been recovered [51]. Among ovicaprids, the sheep far outnumber goats [52]. The ovicaprid exploitation pattern could not be defined based on the Mentesh Tepe bone assemblages, but a study of comparative Neolithic sites did not indicate a specialized exploitation strategy [51].

- **MTT001**, MT342, Grave 342 20 7, 12 individual 1, *Neolithic Shomutepe- Shulaveri culture*. This burial included the remains of individual 1, a 10-14-year-old female, in a Late Neolithic collective grave. The well-preserved skeleton was placed face down, with the legs in a twisted position. This individual was a part of a paleogenetic study and produced genome-wide results [30]. Dating: human tooth, 5879-5562 cal BCE (MAMS-40333, 6802±75 BP) [30].

2.6 Uzun Rama
Country: Azerbaijan
Region: Goranboy district
Coordinates: 40.655752°, 46.579850°
Excavation details: Excavation, Göygöl-Goranboy arxeoloji ekspedisiyası' (B. M.Cəlilov [B. M. Jalilov])

Summary:
The Late Bronze Age burial mounds at Uzun Rama (Uzun Rəmə) are part of a huge cemetery of burial mounds situated at the Uzun Rama limestone plateau at the southern edge of the Kura valley near the city of Ganja [53–57]. They were excavated as part of a salvage archaeology project associated with the extension of the Baku-Tbilisi-Ceyhan pipeline. The plateau is an outlier of the Lesser Caucasus, separated by the Kurekçay river from the northern slopes of the Kara dağ mountain. The village of Qaradağlı is situated about 2-3 km west of the mounds. The plateau is bare of vegetation but is used for the extraction of white lime plaster, which resulted in the destruction of some of the archaeological monuments. The burial field included 205 mounds. Mounds 1, 5, and 8 are associated with the Kura-Araxes/Early Bronze Age culture (ca. 3500-3200 BCE). They contained huge mud brick-built burial chambers with entrance corridors and collective burials of up to 114 individuals. Another group of mounds dates to the Later Bronze/Early Iron Age, c. 1500-1100 BCE. Three of these mounds (Mounds 2-4) were excavated in 2012, including Mound 3, included in this study. Since 2018, an Azeri-Italian project has continued excavation of kurgans at Uzun Rama [58], including more detailed zooarchaeological studies [58], but materials from this later project have not been included in this study.

- **UZR002**, UR/2012/K3, Kurgan 3. *Late Bronze Age culture*. Male. Mound 3 was one of three mounds, oval in shape, with a burial pit covered by river pebbles. Single inhumation. In the mounds, pottery, metal artifacts, and stone (carnelian) beads have been found. Dating: human long bone, no collagen (MAMS-36867; dating this study). Relative dates: ca. 1700-1200 BCE.

3. Archaeological Site and Sample Information for the Oka-Don-Volga Region

3.1 Ksizovo 6
Country: Russian Federation
Region: Zadonsky district, Lipetsk region
Coordinates: 52.279987°, 38.952199°

Excavation details: Excavation by Lipetsk State Pedagogical University, 2004-2005, license №2004-200 and №2005-331 (R.V. Smolyaninov)

Summary:
The site Ksizovo 6 is located in the village of the same name in Zadonsk district, about 60 km southwest of the city of Lipetsk, at the confluence of the Don River to its right tributary, the River Snova. It is situated at the foot of a 20 to 25 m high shore bank of the Don River, on a flat, elevated ground approximately 5.5-6 m above the river level. Ksizovo 6 included both settlement area and burials and was partially (253 km^2) excavated in 2004-2005 [59]. The cultural layers of the settlement range from one to two meters and include material belonging to several cultural phases of the Neolithic-Bronze Age, such as Lyalovo, Middle Don, Lower Don, Sredny Stog, Repin, Catacomb, and Srubnaya [59,60].

Excavators discovered 17 inhumated individuals in 15 flat-earth graves. Anthropological analysis recognized six males, five females, and six children [60]. Based on the radiocarbon dates, the authors of the excavations affiliate the burials to four different archaeological cultures. Most of the burials belong to the Eneolithic Sredny Stog and Neolithic Lyalovo cultures. However, some burials are associated with the Early Neolithic Middle Don culture and the Catacomb culture of the Bronze Age.

- **KSI001**, burial 9, *Middle Don culture*. Burial of a female aged 40-45 years. Associated with the 6th millennium BCE Middle Don Culture [61]. The skeleton was very fragmentary, but the deceased was roughly oriented from northwest to southeast. A bone harpoon, an animal jaw bone, and a canine tooth, along with an accumulation of large limestone fragments, were also found in the grave but did not provide a burial date. According to a radiocarbon date, the burial corresponds to the finds of the Middle Don culture made in the settlement, making it the oldest burial at Ksizovo 6. Dating: human bone 5837-5670 calBCE (Hela-4624, 6870±31 BP; dating this study).

3.2 Rakovka (Bolshaya Rakovka II)
Country: Russian Federation
Region: Krasnoyarsky district, Samara region
Coordinates: 53.693036°, 50.62019°
Excavation details: Excavation by Kuybyshev State Pedagogical Institute (P.P. Varykin, E.V. Kozin)

Summary:
The site is located near the village of Bolshaya Rakovka in the Krasnoyarsk district, about 60 km northeast from the city of Samara, on the floodplain of the Chernovka River and near its confluence with the Sok River, a tributary of the Volga River. This site contains several verified settlement phases, dating from the Early Neolithic (Elshanskaya pottery) to the Late Eneolithic (e.g., Chekalino and Gundorovka types) [62,63]. Excavators discovered one multiple-burial that had been converted into a simple pit and contained a total of 5 individuals. These individuals were placed in extended, south-oriented positions, with skulls and upper parts of the body placed separately in the grave [64]. The lack of any grave inventory leaves open the

question of cultural affiliation, but the fill above the skeletons contained Late Eneolithic pottery.

- **RAV002**, Burial 1, Individual 2, *Late Eneolithic*. Burial of a male and part of a multiple-burial of five individuals. The deceased was placed on his back facing south. Since no artifacts were found in the grave, the cultural context of the burial is not entirely clear. The radiocarbon date of this skeleton (as well as the other individuals) indicates the Eneolithic, the second half of the 4th millennium BCE. Dating: human bone, 3514-3356 calBCE (Hela-4348, 4629±28 BP; dating this study).

3.3 Shagara

Country: Russian Federation
Region: Spas-Klepikovo district, Ryazan region
Coordinates: 55.224822°, 40.111710°
Excavation details: Excavation by State Historical museum since 1986, varying license № 2011-333; 2013-405 (E. D. Kaverzneva)

Summary:
The cemetery is situated on the lower bank of the Shagara lake in the Meshchera lowlands, which is part of the middle Oka river basin at the location of a multi-layer settlement, Shagara I. The settlement includes layers dating from the early Neolithic to the Early Iron Age. To date, a total of 121 graves have been excavated at an area of ca. 1000 m^2 in different stratigraphic and spatial units. The cultural affiliation is based on inventories and burial practices. About a quarter of the graves (n=26) graves date to the mid-4th to mid-3rd millennium BCE (3500-2500 BCE) and are associated with the Eneolithic Volosovo culture, whereas the majority of 87 graves are associated with the early 2nd millennium BC Shagara culture, which dates to approximately 2000-1800 BCE [65] but may extend as early as 2400 BCE. The Volosovo graves cluster in the north-eastern and south-western areas of the burial ground. They comprised single and multiple burials with individuals in crouched positions, generally with the head in the south. Grave inventories included items made of bone and antler, flint and amber, pottery vessels, and in one case, a possible model boat made from clay. The graves included in this study were situated in the central-western and southern clusters of burials. Both belong to the Shagara culture groups.

Bones of wild animals dominate in the faunal assemblages of the settlement layers associated with the two consecutive cultures. About half of the 98.8 % wild species can be attributed to elk (47.8%), followed by beaver (12%), birds (18.1%), and vast amounts of fish bones, including pike, ide, crucian carp, and catfish. The inhabitants' fishing, collecting, and hunting economy are confirmed by stable isotope studies of bone collagen $\delta^{13}C$ and $\delta^{15}N$ values, showing that terrestrial and aquatic foods formed the subsistence basis, with wild herbivores and fish as the primary sources of protein [65].

- **SGR002**, 93A. *Shagara culture*. This burial included three individuals in extended supine positions. Two individuals were placed with heads oriented to the south, while

the third individual was oriented to the north. Individual 1 was male age 45–55, individual 2 was male age 25–35, and individual 3 was female age 50–60. The burial inventory included at least one clay vessel. Dating: human bone, 2572-2350 calBCE (Hela-4715, 3964 ±25 BP; dating this study).

- **SGR005**, 119. *Shagara culture.* This burial included a single individual in an extended supine position with the head to the north. The grave is somewhat set off from the next contemporaneous burial cluster. Dating: human bone, 2031-1893 calBCE (Hela-4718, 3609 ±24 BP; dating this study).

3.4 Vasilevsky Kordon 27

Country: Russian Federation
Region: Dobrovsky district, Lipetsk region
Coordinates: 52.979525°, 40.006380°
Excavation details: Excavation Lipetsk Regional Scientific Public Organization 'Archaeological Research' and Lipetsk State Pedagogical University 2016-2019, license № 2016-433 (R.V. Smolyaninov), №2017-943 (S.V. Shemenev), №2018-938 (A.V. Solovev) and №2019-0576 (M.V. Sultanova).

Summary:
The Eneolithic settlement and cemetery of Vasilyevsky Kordon 27 are located by a lake in the floodplain of the Voronezh River, about 50 km northeast of Lipetsk in the Dobrovsky district. The region is part of the Don Basin and is characterized by a forest-steppe environment. The site was discovered in 2008 and excavated from 2016 to 2019 with an area of 348 m^2. A total of six buildings and four graves were discovered. The site dates to the Eneolithic and Bronze Ages. The majority of the material is associated with the Sredny Stog culture, but the assemblage also includes pottery of Volosovo, Ksizovo, and Catacomb types. A large quantity of flint projectile points and the zooarchaeological material indicate a focus on hunting and fishing at the site [61,66]. Most of the animal bones are represented by elk (60.6%), beaver (16.8%), boar (6.4%), and other wild species. Reptiles such as turtles, mollusks, and fish bones including pike, perch, and catfish, in addition to harpoons and net-sinkers, are indicators for a strong fishing component. The presence of the reptiles suggests seasonal occupation in spring and summer. Few fragments of cattle (1.8%) and possible ovicaprid bones (0.8%) may indicate the first steps of animal husbandry in the Upper Don basin.

All four burials were poorly preserved flat-ground inhumations. Even if artifacts associated with the Volosovo culture were found in some graves, there is very little other material associated with this culture at the site. Therefore, the authors of the excavations connect the burials with the Sredny Stog occupation of the settlement. Radiocarbon dates of human bones from the graves date from the second half of the 5th-millennium BCE until the end of the 4th millennium cal BCE and were evaluated along with the datings of animal bones from the buildings (Sredny Stog) at the site. Nevertheless, two younger dates are from one of the burials (VSK001); considering the aquatic component in the diet, the freshwater reservoir effect is

possible but not studied in this region. The Sredy Stog culture extends to the end of the 4th millennium BCE in this region of the Don basin [67].

- **VSK001**, Burial 1, *Late Sredny Stog culture.* A single inhumation of a male aged 40-50 years in a long oval burial pit in a supine position, the head oriented to the northeast. The inventory included a complete, polished flint axe, a fragment of another adze, a quartzite hammerstone, and a retouched flake. The pit filling contained fragments of Sredny Stog pottery and bones of bear, beaver, wild boar, and elk. The affiliation to the *Sredny Stog culture* is supported by one radiocarbon date, even if there are two much younger radiocarbon dates from the burial. Dating: human bone 3365-3100 calBCE (Hela-4622, 4529±35 BP; dating this study), human bone 1874-1510 calBCE (SPb-2096, 3375±60 BP), animal bone 2135-1770 calBCE (SPb-2100, 3592±55 BP) [68].
- **VSK002**, Burial 3, *Late Sredy Stog culture.* Poorly preserved single inhumation of a female buried in an oval pit in a supine position, the head oriented to the northeast. No burial inventory was detected, but the burial is connected with the *Sredny Stog culture* through radiocarbon dating. Dating: human bone 3649-3528 calBCE (Hela-4623, 4823±31 BP; dating this study).

3.5 Rovenka

Country: Russian Federation
Region: Dobrinsky district, Lipetsk region
Coordinates: 51.946538°, 40.532762°
Excavation details: Excavation Lipetsk Regional Scientific Public Organization Archaeological Research' and Lipetsk State Pedagogical University 2008-2009, license № 2008-1544 (S.V. Uvarkin) and № 2009-31 (A.N. Bessudnov)

Summary:
The burial ground is located in the village of Rovenka in the Dobrinsky district, approximately 100 km southeast of Lipetsk. The site, situated on a promontory overlooking the floodplain of the Bityug River, the left tributary of the River Don, is approximately 5 m above the river level. Altogether eight burials were excavated in 2008-2009, including five adults and three children [69]. Modern land use partially destroyed some burials. However, catacomb structures in some graves, body orientation and position, the use of ochre and charcoal, and grave goods connect the cemetery with the Middle Don Catacomb Culture (2500-2000 BCE) [7].

- **RVK001**, burial 1, *Middle Don Catacomb culture*. Inhumation of a male individual in a rectangular pit. The grave was found in connection to modern land use, which destroyed the lower part of the skeleton from the pelvis down and the forearms. Only the upper part of the body was archaeologically investigated, but judging by the information received from local residents, the body was on the left side with slightly bent legs and hands resting on the knees. A Catacomb Culture clay vessel was found

at the feet, and the upper body and head were covered with ochre. Dating: human bone, 2339-2148 calBCE (MAMS-37453, 3818±18 BP; dating this study).

4. Archaeological Site and Sample Information for the East Urals Region

4.1 Neplyuyevka

Country: Russian Federation
Region: Kartaly district, Chelyabinsk region
Coordinates: 52.88263°, 60.11490°
Excavation details: Excavation Institute of History and Archaeology, Urals Branch of the Russian Academy of Sciences, Ekaterinburg, 2015-2017, license 2015-633, 2016-905, 2017-649 (S.V. Sharapova)

Summary:
The Bronze Age cemetery Neplyuevka is located on the bank of the Yandyrka River (Tobol basin), south of the village. The site consisted of 38 kurgans; during 2015-2017 field campaigns, three kurgans were excavated. Two small kurgans (Kurgans 5 and 9) yielded only non-adult burials [70], while under the mound of the biggest (Kurgan 1), individual primary and double burials of adults and children were recorded [71]. Besides these, cenotaphs and structures for food offerings were placed within the funeral ground. The material analyzed here was sampled from individuals buried in Kurgan 1. Funeral chambers were furnished with stone plates and removed sub-soil clay. This construction was then placed into the lower level of the kurgan mound. A study of the paleosoil supports the hypothesis that the burial area initially functioned as a flat burial ground and was later covered with a kurgan mound [72]. The most impressive interments, marked with rock-clay funeral expenditure, were mapped outside the geometrical center of the kurgan and contained male and female skeletal remains. There is no specific order for the location of the juvenile burials. Double burials occurred among all age categories, i.e., juveniles, young adults, and older adults. The set of grave goods includes votive ceramic vessels (none of which appear to have been used), rings, bracelets made of bronze, and bronze temporal pendants plated with golden foil. Graves 5 and 9 from Kurgan 1 are associated with the Srubnaya-Alakul culture [73], which was present in the region from ca. 1900-1600 BCE.

- **NEP008**, K1 B5, Kurgan 1, Grave 5. *Srubnaya-Alakul culture.* 30-40-year-old female. Dating: relative ca. 2030-1740 BCE.
- **NEP013**, K1 B9 SK1, Kurgan 1, Grave 9, Skeleton 1, *Srubnaya-Alakul culture.* This burial included at least 2 adult individuals. Skeleton 1 was a male, over 50-year-old. The second individual was possibly a young female. The grave was heavily disturbed by tunneling animals (i.e., rodents). Dating: relative ca. 2030-1740 BCE.

References

1 Korenevskiy, S.N., Kalmykov, A.A. Maikop graves from burial mound 22 of the Aigursky 2 cemetery [in German]. *Rossiyskaya Arkheologiya* **4**, 77–94 (2017).

2 Gerasimova, M.M., Pezhemskiy, D.V., Jablonskiy, L.T. Paleoantropologicheskie materialy maykopskoy epokhi iz Zentralnogo Predkavkazya. *Materialy po Izucheniyu IstorikoKulturnogo Naslediya Severnom Kavkaza*, 91–121 (2007).

3 Hollund, H.I., Higham, T., Belinskij, A. Investigation of palaeodiet in the North Caucasus (South Russia) Bronze Age using stable isotope analysis and AMS dating of human and animal bones. *J Archaeol Sci* **37**, 2971-2983 (2010).

4 Wang, C-C., Reinhold, S., Kalmykov, A., Wissgott, A., Brandt, G., Jeong, C. *et al.* Ancient human genome-wide data from a 3000-year interval in the Caucasus corresponds with eco-geographic regions. *Nature Communications* **10**, 590 (2019).

5 Korenevskij, S.N., Belinskij, A.B., Kalmykov, A.A. *Bol'šoj Ipatovskij kurgan na Stavropol'e kak arheologičeskij istočnik po èpohe bronzovogo veka na stepnoj granice Vostočnoj Evropy i Kavkaza* (Nauka, 2007).

6 Belinskij, A. & Kalmykov, A. in *Rad und Wagen: der Ursprung einer Innovation; Wagen im Vorderen Orient und Europa* (eds Fansa, M. & Burmeister, S.) 201-200 (Philipp von Zabern, 2004).

7 Knipper, C., Reinhold, S., Gresky, J., Berezina, N., Gerling, C., Pichler, S.L. *et al.* Diet and subsistence in Bronze Age pastoral communities from the southern Russian steppes and the North Caucasus. *PLoS One* **15**, e0239861 (2020).

8 Key, F.M., Posth, C., Esquivel-Gomez, L.R., Hübler, R., Spyrou, M.A., Neumann, G.U. *et al.* Emergence of human-adapted Salmonella enterica is linked to the Neolithization process. *Nat Ecol Evol* **4**, 324–333 (2020).

9 Korenevskiy, S.N. Utochneniye kolonki radiouglerodnykh dat Bol'shogo Ipatovskogo kurgana i osobennostey simvoliki yego konstruktsii. *Kratkiye soobshcheniya Instituta arkheologii* **236**, 42–49 (2014).

10 Reinhold, S., Belinskij, A.B., Atabiev, B.H. in *Der Kaukasus zwischen Osteuropa und Vorderem Orient: Dialog der Kulturen, Kultur des Dialoges* (eds Kašuba, M.T., Reinhold, S., & Piotrovskij, J.J.) 105-172 (Dietrich Reimer Verlag, 2020).

11 Nagler, A., Uhl, R. in *Current Research in Eurasia* (ed Hansen S) 60-61 (Eurasien-Abteilung des Deutschen Archäologischen Instituts, 2017).

12 Berezin, Y.B., Kalmykov, A.A. A burial mound near the village of Krasnogvardeyskoye, Stavropol Territory [in Russian]. *Materials on the study of historical and cultural heritage of the North Caucasus* **1**, 55–95 (1998).

13 Kantorovich, A.R., Maslov, V.E., Petrenko, V.G. Pogrebeniya maykopskoy kultury kurgana No. 1 mogilnika Marinskaya 5. *Materialy po izucheniyu istoriko-kulturnogo naslediya Severnogo Kavkaza* **10**, 71–108 (2013).

14 Reinhold, S., Gresky, J., Berezina, N., Kantorovich, A.R., Knipper, C., Maslov, V.E. *et al.* in *Appropriating innovations: Entangled Knowledge in Eurasia, 5000–1500 BCE* (eds Stockhammer, P. & Maran, J.) 78-97 (Oxbow Books Limited, 2017).

15 Myachin, S.V. *Protection-rescue excavations of kurgan cemeteries Tonnel'nyy-4, Tonnel'nyy-5, Tonnel'nyy-7, Nevinnomysskiy-4, Nevinnomysskiy-3, Nevinnomysskiy-2, Nevinnomysskiy-1, Nadzornyy-1 in Kochubeevskiy district, Stavropol' region in 2012.* (Institute of Archaeology of the Russian Academy of Sciences, 2013).

16 Kalmykov, A.A., Berezina, N.J., Greski, Y., Dobrovolskaya, M.V., Buzhilova, A.P. The burial of the master caster of the Lola culture in Stavropol Territory [in Russian]. *Brief communications of the Institute of Archaeology* **251**, 64–79 (2018).

17 Korenevsky, S.N., Berezina, N.J., Berezin, Y.B., Greski, Y. in *The Caucasus Mountains and the Mesopotamian Steppe at the Dawn of the Bronze Age: Dedicated to the 90th anniversary of RA Munchayev* (ed Amirkhanov, H.A.) 155-178 (IA RAS, 2019).

18 Gresky, J., Batieva, E., Kitova, A., Kalmykov, A., Belinskiy, A., Reinhold, S. *et al.* New cases of trepanations from the 5th to 3rd millennia BC in Southern Russia in the context of previous research: Possible evidence for a ritually motivated tradition of cranial surgery? *Am J Phys Anthropol* **160**, 665–682 (2016).

19 Bohn, U., Neuhäusl, R., Gollub, G., Hettwer, C., Neuhäuslová, Z., Raus, T. *et al. Karte der natürlichen Vegetation Europas/Map of the natural vegetation of Europe.* (Bundesamt für Naturschutz, 2003).

20 Andrades Valtueña, A., Mittnik, A., Key, F.M., Haak, W., Allmäe, R., Belinskij, A. *et al.* The Stone Age Plague and Its Persistence in Eurasia. *Curr Biol* **27**, 3683–3691, 2017.

21 Govedarica B. *Zepterträger – Herrscher der Steppen Die frühen Ockergräber des älteren Äneolithikums im karpatenländischen Gebiet und im Steppenraum Südost- und Osteuropas* (Philipp von Zabern, 2004).

22 Tucker, K., Berezina, N., Reinhold, S., Kalmykov, A., Belinskiy, A., Gresky, J. An accident at work? Traumatic lesions in the skeleton of a 4th millennium BCE 'wagon driver' from Sharakhalsun, Russia. *Homo* **68**, 256–273 (2017).

23 Knipper, C., Reinhold, S., Gresky, J., Belinskiy, A., Alt, K.W. in *Isotopic Investigations of Pastoralism in Prehistory* (eds Kristiansen, K., Bánffy, E., Attema, P., Ventresca Miller, A.R., Makarewicz, C.A.) 123-140 (Routledge, 2017).

24 Korenevsky, S.N., Rezepkin, A.D. Radiocarbon chronology of sites of the type of Maykop burial and Novosvobodnensk tombs [in Russian]. *Problemy istorii, filologii, kul'turi* **22**, 109–127 (2008).

25 Mimokhod, R.A. *Lola Culture: The northwestern Caspian region at the turn of the Middle and Late Bronze Age* [in Russian] (IA RAN, 2013).

26 Akhundov, T. Alkhantepe - a settlement of the early Bronze Age in Azerbaijan [in Russian]. *Notes of the IIMK RAS* **10**, 78–92 (2014).

27 Akhundov, T.I. Archaeological Sites of the Mugan Steppe and Prerequisites for

Agricultural Settlement in the South Caucasus in the Neolithic-Eneolithic. *Stratum Plus* **2**, 219-236 (2011).

28 Ollivier, V., Fontugne, M., Lyonnet, B., Chataigner, C. Base level changes, river avulsions and Holocene human settlement dynamics in the Caspian Sea area (middle Kura valley, South Caucasus). *Quaternary International* **395**, 79–94 (2016).

29 Taghiyeva, Y.N. in *Understanding the Problems of Inland Waters: Case Study for the Caspian Basin (UPCB)*. 289-292 (Institute of Geography of Azerbaijan National Academy of Sciences, 2018).

30 Skourtanioti, E., Erdal, Y.S., Frangipane, M., Balossi Restelli, F., Yener, K.A., Pinnock, F. *et al.* Genomic History of Neolithic to Bronze Age Anatolia, Northern Levant, and Southern Caucasus. *Cell* **181**, 1158–1175.e28 (2020).

31 Quliyev, F.E., Qasımov, P.P., Ələkbərov, V.Ə., Səlimbəyov, Ş.A, Məmmədov, Y.V. in *Archaeological research in Azerbaijan - 2015-2016* (ed Rəhimova, M.N.) 62-75 (Institute of Archeology and Ethnography of AMEA, 2017).

32 Nishiaki, Y., Guliyev, F (eds). *Göytepe: Neolithic Excavations in the Middle Kura Valley, Azerbaijan* (Archaeopress: 2020).

33 Gasimov, P. The burial items-replicas of funerary tradition of the Khojaly-Gadabay culture [in Azerbaijani]. *Azerbaijan Archaeology* **20**, 22–37 (2017).

34 Karimikiya, A., Rezaloo, R. An introduction to the culture of Khojaly-Gadabay in the South Caucasus and its expansion in the northwest of Iran. *Journal of Archaeological Studies* 12, 217-241 (2020).

35 Hasanov, Z.H. Arrowheads of the Early Scythian Period in the Eastern Part of the Caucasus: Scythian migration problem. *Stratum Plus* **3**, 17–50 (2017).

36 Eminli, J. in *From Albania to Arrān* (ed Hoyland, R.) 277-304 (Gorgias Press, 2020).

37 Eminli, J. in *The Gabala Archaeological Expedition: Reports, Findings,* 74–107 (CBS, 2013).

38 Eminli, J., Iskandarov, E. in *The Gabala Archaeological Expedition: Reports, Findings,* 102–127 (CBS, 2016).

39 Eminli J, Iskandarov E. in *The Gabala Archaeological Expedition: Reports, Findings 2015-2016*, 156–217 (CBS, 2017).

40 Babaev, I. *Cities of Caucasian Albania from the 4th c. BCE to 3rd c. CE* (Elm,1990).

41 Vakhidov, R.M. *Mingecevir in the 3rd-8th centuries* (Academy of Sciences AzSSR, 1961).

42 Mustafaev, I.D. *On the history of the Cultivation of Grain Crops in Azerbaijan*. (Academy of Sciences AzSSR, 1955).

43 Baxsəliyev, V., Ristvet, L., Gopnik, H., Swerida, J., Nugent, S. Qızqalası Yas¸ayıs¸

Yerində 2015-ci Ildə Aparılan Arxeoloji Aras¸dırmalar. *Azərbaycan MEA-nın Xəbərləri Ictimai elmlər seriyası* **2**, 178–198 (2016).

44 Gopnik, H. Grounded: A Late Bronze Age fortress on the Şərur Valleyfloor, Naxçıvan. Conference presentation at the *81st Annual Meeting of the Society for American Archaeology*, Orlando, FL, tDAR 404891 (2016).

45 Hammer, E. Highland fortress-polities and their settlement systems in the southern Caucasus. *Antiquity* **88**, 757–774 (2014).

46 Nugent, S.E. *Pastoral Mobility and the Formation of Complex Settlement in the Middle Bronze Age Serur Valley, Azerbaijan*. PhD Disseration (Ohio State University, 2017).

47 Nugent, S.E. Pastoralism and Emergent Complex Settlement in the Middle Bronze Age, Azerbaijan: isotopic analyses of mobility strategies in transformation. *Am J Phys Anthropol* **171**,120–141 (2020).

48 Lau, H., Proctor, L., Gopnik, H., Bakhshaliyev, V. Agropastoralism in Middle Bronze through Early Iron Age Naxçıvan: Zooarchaeological and paleoethnobotanical data from Qızqala. *Journal of Archaeological Science: Reports* **33**, 102535 (2020).

49 Swerida, J., Nugent, S. in *Fashioned Selves: Dress and Identity in Antiquity* (ed Cifarelli, M.) 11-26 (Oxbow Books, 2019).

50 Pecqueur, L., Jovenet, E., Abadie, I., Ringenbach, C. in *The Kura Projects. New Research in the Later Prehistory of Southern Caucasus* (eds Helwing, B., Aliyev, T., Lyonnet, B., Guliyev, F., Hansen, S., Mirtskhulava, G.) 163-178 (Dietrich Reimer Verlag, 2017).

51 Benecke, N. Exploitation of animal resources in Neolithic settlements of the Kura Region (South Caucasia). in *The Kura projects: New research on the later prehistory of the southern Caucasus Archeology in Iran and Turan* (eds Helwing, B., Aliyev, T., Lyonnet, B., Guliyev, F., Hansen, S., Mirtskhulava, G.) 357-369 (Dietrich Reimer Verlag, 2017).

52 Herrscher, E., Poulmarc'h, M., Pecqueur, L., Jovenet, E., Benecke, N., Decaix, A. *et al.* Dietary inferences through stable isotope analysis at the Neolithic and Bronze Age in the southern Caucasus (sixth to first millenium BC, Azerbaijan): From environmental adaptation to social impacts. *Am J of Phys Anthropol* **167**,856–875 (2018).

53 Jalilov, B. in *Azərbaycanda arxeoloji tedqiqatlar*, 94-101 (Xezer Universiteti, 2010).

54 Jalilov, B. in *Azərbaycanda arxeoloji tedqiqatlar*, 95–102 (Xezer Universiteti, 2011).

55 Jalilov, B. in *Azərbaycanda arxeoloji tedqiqatlar*, 128-137 (Xezer Universiteti, 2012).

56 Jalilov, B. in *Azərbaycanda arxeoloji tedqiqatlar*, 128-137 (Xezer Universiteti, 2013).

57 Modwene, P.H., Pecqueur, L., Jalilov, B. An overview of Kura-Araxes funerary practices in the Southern Caucasus. *Paléorient* **40**, 231–246 (2014).

58 Laneri, N., Jalilov, B., Crescioli, L., Guarducci, G., Kneisel, J., Poulmarch, M. *et al.*

GaRKAP 2018: The first season of the Azero-Italian Ganja Region Kurgan Archaeological Project in Western Azerbaijan. *Ancient Near Eastern Studies* **56**, 135-162 (2019).

59 Lavrushin, Y.A., Cpiridonova, E.A., Bessudnov, A.H., Smolyaninov, R.V. *Natural disasters in the Holocene of the Upper Don basin* (GEOS, 2009).

60 Vasilyev, S.V., Smolyaninov, R.V., Borutskaya, S.B., Bessudnov, A.N. Neolithic-Eneolithic Population of the Upper Don Region and its Burial Customs (by the materials of the burial ground Ksizovo 6). *Stratum Plus* **2**, 167–195 (2018).

61 Smolyaninov, R., Skorobogatov, A., Surkov, A. Chronology of Neolithic sites in the forest-steppe area of the Don River. *Documenta Praehistorica* **44**, 192–203 (2017).

62 Shalapinin, A.A. The Late Chalcolithic Ceramics of the Volga Forest-Steppe. *Archaeology, Ethnography, and Anthropology of Eurasia* **46**, 27–33 (2018).

63 Andreev, K.M., Vybornov, A.A., Kulkova, M.A., Khramov, D.Y. Absolute Chronology of Ceramics of the Lugovskoy Type. *Samara Scientific Bulletin* **8**, 132–135 (2019).

64 Barynkin, P.P., Kozin, E.V. in *Antiquities of the East European Forest Steppe* (ed Merpert, N.Y.) 94-119 (SamGPI, 1991).

65 Shishlina, N., Kaverzneva, E., Fernandes, R. Subsistence strategies of Meshchera lowlands populations during the Eneolithic period–The Bronze Age: Results from a multidisciplinary approach. *Journal of Archaeological Science: Reports* **10**, 74–81 (2016).

66 Smolyaninov, R.V., Yurkina, E.S., Yanish, Y.Y., Zheludkov, A.S., Shemeniov, S.V., Soloviev, A.V. The Eneolithic settlement and burial site Vasilyevskiy Kordon 27: evidence of hunting and fishing (excavations 2016-2018, preliminary publication). *Samara Journal of Science* **8**, 122–130 (2019).

67 Skorobogatov, A., Smolyaninov, R. in *Radiocarbon in Archaeology and Paleoecology: past, present, and future* (eds Burova, N.D., Vybornov, A.A., Kulkova, M.A.) (Porto-Print, 2020).

68 Smolyaninov, R.V., Bessudnov, A.N., Zheludkov, A.S., Kulichkov, A.A., Yurkina, E.S., Yanish, E.Y. in *Upper Don Archaeological Collection* (ed Bessudnov, A.N.) 229-244 (Lipetsk State Pedagogical University, 2017).

69 Ivashov, M.V. *Monuments of the Catacomb Period in the Upper Don*. PhD Dissertation (Southern Federal University Voronesh, 2014).

70 Loyer, J., Sharapova, S.V. Paleopathological study of the bronze age children's burials (the Neplujevsky cemetery case study). *Ural Historical Bulletin* **54** 103–112 (2017).

71 Karapetian, M.K., Sharapova, S.V., Yakimov, A.S. New data on lifestyle of the population during the Bronze Age in the southern Trans-Urals. *Ural Historical Bulletin* **62**, 28–37 (2019).

72 Kuptsova, L.V. in *Archaeological Monuments of the Orenburg Region* (ed Morgunova,

N.L.) 177-195 (Orenburg State Pedagogical University, 2014).

73 Krzewińska, M., Kılınç, G.M., Juras, A., Koptekin, D., Chyleński, M., Nikitin, A.G. *et al.* Ancient genomes suggest the eastern Pontic-Caspian steppe as the source of western Iron Age nomads. *Sci Adv* **4**: eaat4457 (2018).
