# Supplementary material for: Emergence and intensification of dairying in the Caucasus and Eurasian steppes
Source: Nat Ecol Evol. 2022 Apr 7;6(6):813–22. doi: 10.1038/s41559-022-01701-6 (PMC9177415; doi:10.1038/s41559-022-01701-6)
Supplement: Supplementary file 2 — Reporting Summary [file 41559_2022_1701_MOESM2_ESM.pdf]

nature portfolio

Corresponding author(s): Christina Warinner

Last updated by author(s): Jan 24, 2022

Reporting Summary

Nature Portfolio wishes to improve the reproducibility of the work that we publish. This form provides structure for consistency and transparency in reporting. For further information on Nature Portfolio policies, see our Editorial Policies and the Editorial Policy Checklist.

Statistics

For all statistical analyses, confirm that the following items are present in the figure legend, table legend, main text, or Methods section.

n/a	Confirmed	
☐	☒	The exact sample size (*n*) for each experimental group/condition, given as a discrete number and unit of measurement
☐	☒	A statement on whether measurements were taken from distinct samples or whether the same sample was measured repeatedly
☒	☐	The statistical test(s) used AND whether they are one- or two-sided<br>*Only common tests should be described solely by name; describe more complex techniques in the Methods section.*
☐	☒	A description of all covariates tested
☐	☒	A description of any assumptions or corrections, such as tests of normality and adjustment for multiple comparisons
☐	☒	A full description of the statistical parameters including central tendency (e.g. means) or other basic estimates (e.g. regression coefficient) AND variation (e.g. standard deviation) or associated estimates of uncertainty (e.g. confidence intervals)
☒	☐	For null hypothesis testing, the test statistic (e.g. *F*, *t*, *r*) with confidence intervals, effect sizes, degrees of freedom and *P* value noted<br>*Give P values as exact values whenever suitable.*
☒	☐	For Bayesian analysis, information on the choice of priors and Markov chain Monte Carlo settings
☒	☐	For hierarchical and complex designs, identification of the appropriate level for tests and full reporting of outcomes
☒	☐	Estimates of effect sizes (e.g. Cohen's *d*, Pearson's *r*), indicating how they were calculated

Our web collection on statistics for biologists contains articles on many of the points above.

Software and code

Policy information about availability of computer code

Data collection	Xcaliber (ThermoFisher Scientific)
Data analysis	Mascot (Matrix Science, v 2.6.0); MSConvert v 3.0.11781; Scaffold (Proteome Software, v 4.9.0); OxCal v 4.4 with the IntCal20 atmospheric curve.

For manuscripts utilizing custom algorithms or software that are central to the research but not yet described in published literature, software must be made available to editors and reviewers. We strongly encourage code deposition in a community repository (e.g. GitHub). See the Nature Portfolio guidelines for submitting code & software for further information.

Data

Policy information about availability of data

All manuscripts must include a data availability statement. This statement should provide the following information, where applicable:

- Accession codes, unique identifiers, or web links for publicly available datasets
- A description of any restrictions on data availability
- For clinical datasets or third party data, please ensure that the statement adheres to our policy

Raw data files are available through the ProteomeXchange Consortium via the PRIDE partner repository under accession PDX027728.

Field-specific reporting

Please select the one below that is the best fit for your research. If you are not sure, read the appropriate sections before making your selection.

☐ Life sciences ☐ Behavioural & social sciences ☒ Ecological, evolutionary & environmental sciences

For a reference copy of the document with all sections, see nature.com/documents/nr-reporting-summary-flat.pdf

Ecological, evolutionary & environmental sciences study design

All studies must disclose on these points even when the disclosure is negative.

Study description	This study conducted shotgun tandem mass spectrometry on proteins present in human dental calculus obtained from 29 archaeological sites in Russia and Azerbaijan. Proteins were identified using Mascot and validated using Scaffold, and dietary proteins were analyzed to reconstruct past dairy pastoralism strategies.
Research sample	46 human dental calculus specimens were analyzed from 45 individuals at 29 archaeological sites. A comprehensive overview of these specimens is provided in Dataset S1, and detailed osteological and archaeological context data is provided in the SI.
Sampling strategy	We sought to comprehensively sample archaeological sites relevant for the research question. Dental calculus was collected from individuals for whom at least 5 mg of dental calculus was available, but care was taken to avoid sampling individuals with small amounts of calculus, for whom this collection would represent sample exhaustion.
Data collection	Proteomics data was collected using a Q-Exactive mass spectrometer (Thermo Scientific, Bremen, Germany) coupled to an ACQUITY UPLC M-Class system (Waters AG, Baden-Dättwil, Switzerland) at the Functional Genomics Center Zurich of the University/ETH Zurich. AMS radiocarbon dating was performed at the Curt-Engelhorn-Zentrum Archäometrie (CEZA) in Mannheim, Germany, the Finnish Museum of Natural History (Hela) in Helsinki, Finland, the Oxford Radiocarbon Accelerator Unit (OxA) in Oxford, UK, the Kiev Radiocarbon Laboratory (KI) in Kiev, Ukraine, Russian Academy of Sciences (GIN) in Moscow, Russia.
Timing and spatial scale	Samples were analyzed over the period 2016-2021.
Data exclusions	Data generated during a QC-failed instrument run (failure to achieve chromatographic separation on the UPLC prior to analysis) were discarded and the experiment repeated. One collected sample was excluded from the study after a labeling error was discovered that made it its archaeological provenance uncertain.
Reproducibility	Sample ZO002 was analyzed twice, yielding highly similar results.
Randomization	NA
Blinding	NA

Did the study involve field work? ☐ Yes ☒ No

Reporting for specific materials, systems and methods

We require information from authors about some types of materials, experimental systems and methods used in many studies. Here, indicate whether each material, system or method listed is relevant to your study. If you are not sure if a list item applies to your research, read the appropriate section before selecting a response.

Materials & experimental systems

n/a	Involved in the study	
☒	☐	Antibodies
☒	☐	Eukaryotic cell lines
☐	☒	Palaeontology and archaeology
☒	☐	Animals and other organisms
☒	☐	Human research participants
☒	☐	Clinical data
☒	☐	Dual use research of concern

Methods

n/a	Involved in the study	
☒	☐	ChIP-seq
☒	☐	Flow cytometry
☒	☐	MRI-based neuroimaging

Palaeontology and Archaeology

Specimen provenance	Various archaeological sites in Russia and Azerbaijan; see Data S1 and the SI for a comprehensive list of archaeological sites, locations, and permit license numbers.
Specimen deposition	Contact information for each specimen is provided in the SI.

Dating methods

New radiocarbon dates and lab codes are provided in Data S1.

☒ Tick this box to confirm that the raw and calibrated dates are available in the paper or in Supplementary Information.

Ethics oversight

Study consists of the analysis of previously excavated archaeological remains, which are exempt from human subjects ethical review; excavation partners providing specimens are coauthors on the study and excavation licenses are provided in the SI.

Note that full information on the approval of the study protocol must also be provided in the manuscript.
